# The Association Between Physical Activity Level and Severity of Knee Osteoarthritis: A Single Centre Study in Saudi Arabia

**DOI:** 10.7759/cureus.24377

**Published:** 2022-04-22

**Authors:** Abdulrahman A Aldosari, Saeed Majadah, Khaled A Amer, Hend H Alamri, Rawan N Althomali, Reemah F Alqahtani, Rahaf A Alamer, Shuruq Z Alshehri, Roqayya M Alhayyani, Shahenda Y Aleman, Mansour Somaily

**Affiliations:** 1 College of Medicine, King Khalid University, Abha, SAU; 2 Department of Medicine, Rheumatology Division, Khamis Mushayt General Hospital, Abha, SAU; 3 Family Medicine, Family Medicine Joint Program, Abha, SAU; 4 Department of Medicine, Rheumatology Division, King Khalid University, Abha, SAU

**Keywords:** saudi arabia, effect, relations, oa severity, physical activity, knee osteoarthritis

## Abstract

Background and aim

Knee osteoarthritis (OA) is a disabling joint disease that results in degeneration of the joint cartilage. Many studies demonstrate the risk factors and complications of knee osteoarthritis, but how physical activity impacts the osteoarthritis severity needs to be studied. The study aimed to assess the impact and association of physical activity level with the severity of knee osteoarthritis among patients attending a single center in the Aseer region, southwest Saudi Arabia.

Methods

An analytical cross-sectional study was conducted targeting all patients clinically diagnosed with knee osteoarthritis in the Aseer region from June 2021 to September 2021. Data was collected using a pre-structured online questionnaire. The questionnaire included patients' socio-demographic data and clinical data on knee osteoarthritis. The level of physical activity was assessed using the International Physical Activity Questionnaire (IPAQ). The severity of knee osteoarthritis was assessed using the index of severity for osteoarthritis of the knee.

Results

Out of 473 patients clinically diagnosed with knee OA, only 145 patients met our inclusion criteria. Patients' ages ranged from 35 years to 71 years, with a mean age of 44.3 (±12.9) years. A total of 122 (84.1%) patients were females. Sixty-five (44.8%) patients complained of knee OA for less than two years, 46 (31.7%) for two to five years, and 34 (23.4%) were diagnosed for more than five years. A total of 96 (66.2%) had a low level of physical activity, 32 (22.1%) had a moderate level of physical activity, while 17 (11.7%) had a high level of physical activity. Thirty-eight (26.2%) cases complained of extremely severe knee OA, 37 (25.5%) had very severe knee OA, 28 (19.3%) complained of severe knee OA, 36 (24.9%) had mild to moderate knee OA, while six (4.1%) had minimal knee OA. There was a significant beneficial effect of physical activity on the severity of knee OA.

Conclusion

The study revealed that physical activity, especially at high levels, was associated with lower knee OA severity which means a beneficial effect. Most of the patients included in the study had severe to extremely severe knee OA with low physical activity levels.

## Introduction

Knee osteoarthritis (OA) is a frequent progressive joint disorder that is featured by chronic pain with functional debility [1]. Globally, knee OA is responsible for nearly 80% of the burden of OA, which is mainly associated with obesity and age [2]. Until now, no effective treatment for knee OA is available except for knee arthroplasty, which is considered the main treatment at a progressive stage of the disease, but with a high economic burden due to its associated costs [3]. The research found that the worldwide incidence of knee OA was 203 per 10,000 person-years (95% CI: 106-331) in individuals aged 20 and over. Similarly, there are around 86.7 (95% CI: 45.3-141.3) million individuals (20 years and older) annually with knee OA in 2020 worldwide [4,2].

Physical activity's effect on the joint is controversial, with either injurious or beneficial effects on the joints [5]. The injurious effect of severe physical activities on articular cartilage is well documented, while there is less evidence of the valuable effect of recreational or moderate physical exercise on the joints; likewise, in the Framingham cohort study, the increased risk of knee osteoarthritis was not reported among those practicing habitual or light or moderate physical activity [6]. 

Recently, physical activity guidelines recommended at least 150 minutes of moderate or 75 minutes of severe physical activity per week in at least 10 minutes sessions [7]. Guiding patients with OA to improve their physical life, and helping the patient by arranging exercise therapy or physiotherapy, is vital, as most of those cases are not adherent to physical activity guidelines, with lower activity than their age-matched pioneers* *[8]. Also, physical inactivity among cases with OA may put them at high risk for other comorbidities and functional deterioration, leading to higher health care costs. Furthermore, a study shows that doing physical activity such as ambulation for 150 minutes per week might not be tolerable for individuals with very severe knee OA; thus, other types of physical activity might be recommended for them, such as biking and walking including walking stairs​​​​ [9]. The current study was conducted to assess the level of physical activity in patients with knee osteoarthritis and to assess the impact of physical activity level on the severity of knee osteoarthritis.

## Materials and methods

An analytical cross-sectional study was conducted targeting all patients clinically diagnosed with knee osteoarthritis attending the orthopedic and rheumatology clinics at Khamis Mushayt General Hospital from June to September 2021. The Research Ethics Committee of Bisha University approved the study (number: H-06-BH-087). Patients aged less than 35 years or diagnosed with osteoarthritis other than knee osteoarthritis, such as hip osteoarthritis or ankle osteoarthritis, patients with a medical condition that might affect the physical activity other than osteoarthritis or rheumatoid arthritis-like osteoporosis or fractures, were excluded.

The questionnaire elements were assessed and reviewed by a panel consisting of orthopedics and Arabic language translators to ensure the content validity and accuracy of the translation process. We assessed the reliability of the questionnaire items on 15 patients during a pilot study. The questionnaire included patients' socio-demographic data like age, gender, job category, co-morbidities, and clinical data of knee osteoarthritis, including duration since diagnosis, received treatment, analgesics intake, history of surgical repair, and physiotherapy. The level of physical activity was assessed using the International Physical Activity Questionnaire (IPAQ) [1]. IPAQ was summed for each physical activity domain to estimate the total time spent on occupational, transportation, household, and leisure-related physical activities, as well as the total time. Data from the short IPAQ was summarized according to the recorded physical activities (walking, moderate, and vigorous activities) and estimated time spent sitting per week. Both the short and long-form data were used to estimate total weekly physical activity by weighting the reported minutes per week for each activity category by a metabolic rate energy (MET) expenditure estimate assigned to each category of activity. MET levels were obtained from the compendium of 2,000 physical activities, including moderate-intensity activities between three and six METs and high activities as ≥6 METs [10,11]. The severity of knee osteoarthritis was assessed using the index of severity for osteoarthritis of the knee [12]. The questionnaire was given for fulfillment to patients who were clinically diagnosed with knee osteoarthritis after explaining the purpose and confirming the confidentiality of the data. 

Data analysis

After data was extracted, it was revised, coded, and analyzed with statistical software IBM SPSS version 22 (IBM Inc., Armonk, USA). All statistical analysis was done using two-tailed tests. A p-value less than 0.05 was considered as statistically significant. Descriptive analysis based on the frequency and percent distribution was done for all variables, including participants' socio-demographic data, including gender, job, and co-morbidities. Also, clinical data regarding knee osteoarthritis, including duration and medications besides knee surgery and trauma, were tabulated. The patient's level of physical activity and severity of knee osteoarthritis were also tabulated and graphed. Cross-tabulation was used to assess the distribution of knee osteoarthritis severity by patient's bio-demographic data and to test the association between physical activity level and severity of knee osteoarthritis. Relationships were tested using the Pearson chi-square test and exact probability test for small frequency distributions.

## Results

Out of 473 patients clinically diagnosed with knee osteoarthritis, only 145 patients met our inclusion criteria. Patients' ages ranged from 35 years to 71 years, with a mean age of 44.3 (±12.9) years. A total of 122 (84.1%) patients were females, and 68 (46.9%) were active in their jobs, while 62 (42.8%) had no job. Regarding co-morbidities, 37 (25.5%) complained of rheumatoid arthritis, 29 (20%) were diabetic, and 29 (20%) were hypertensive, as shown in Table 1.

**Table 1:** Bio-demographic data of patients with knee osteoarthritis DM - diabetes mellitus; HTN - hypertension

Bio-demographic data	No	%
Age in years		
35-40	57	39.30%
41-45	54	37.20%
46-55	7	4.80%
56-65	16	11.00%
>65	11	7.60%
Gender		
Male	23	15.90%
Female	122	84.10%
Job		
No job	62	42.80%
Active	68	46.90%
Retired	15	10.30%
Co-morbidities		
None	65	44.80%
DM	29	20.00%
Rheumatoid arthritis	37	25.50%
Autoimmune disease	1	0.70%
HTN	29	20.00%
Cardiac disease	3	2.10%
Others	15	10.30%

Table 2 shows that 65 (44.8%) patients complained of knee osteoarthritis for less than two years, 46 (31.7%) from two to five, and 34 (23.4%) were diagnosed for more than five years. A total of 70 (48.3%) used medications for osteoarthritis; 36 (51.4%) of them used medication daily, 14 (20%) used medications several times a week, and 12 (17.1%) used only when needed. Forty (27.6%) received paracetamol, 38 (26.2%) received nonsteroidal anti-inflammatory drugs (NSAIDs), while 48 (33.1%) did not use painkillers. Only nine (6.2%) patients had undergone knee replacement surgery and 27 (18.8%) visited the physiotherapist frequently.

**Table 2:** Clinical data of knee osteoarthritis among the study patients KOA - knee osteoarthritis; NSAIDs - nonsteroidal anti-inflammatory drugs

KOA clinical data	No	%
Since when were you diagnosed with knee osteoarthritis?		
<2 years	65	44.80%
2-5 years	46	31.70%
>5 years	34	23.40%
Do you use any medications for osteoarthritis?		
Yes	70	48.30%
No	75	51.70%
How frequently do you use the medication?		
Every day	36	51.40%
Several times a week	14	20.00%
Once a week	6	8.60%
Several times a month	2	2.90%
When needed only	12	17.10%
Do you use any of the following analgesics?		
Do not use painkillers	48	33.10%
NSAIDs	38	26.20%
Other	19	13.10%
Paracetamol (Adol® or similar)	40	27.60%
Have you ever undergone knee replacement surgery?		
Yes	9	6.20%
No	136	93.80%
Are you visiting the physiotherapist frequently?		
Yes	27	18.80%
No	117	81.30%

**Figure 1:**
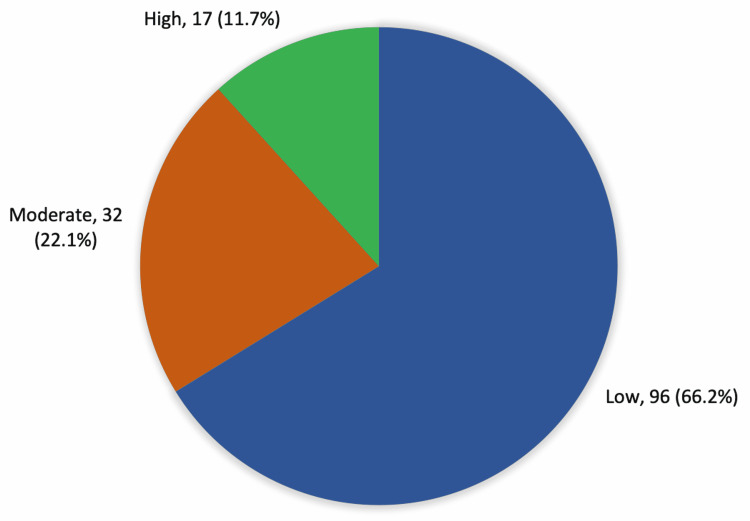
The level of physical activity among patients with knee osteoarthritis among the study patients

Figure 1 presents that 96 (66.2%) of patients had a low level of physical activity, 32 (22.1%) had a moderate level of physical activity and 17 (11.7%) had a high level of physical activity.

**Figure 2:**
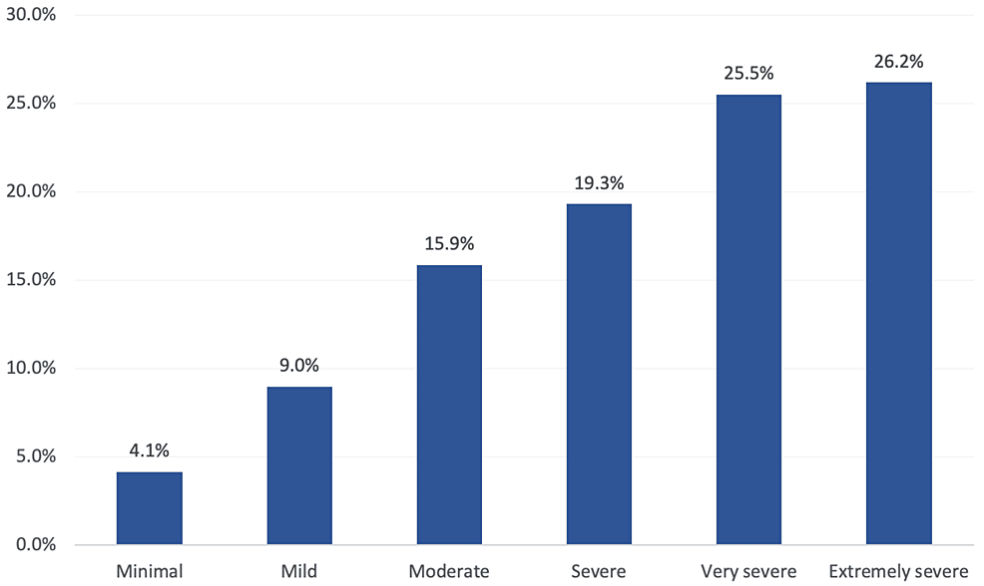
The severity of knee osteoarthritis among the study patients

A total of 38 (26.2%) patients complained of extremely severe knee OA, 37 (25.5%) had very severe knee OA, 28 (19.3%) complained of severe knee OA, 36 (24.9%) had mild to moderate knee OA, while six (4.1%) had minimal OA as shown in Figure 2.

Table 3 reports that very/extremely severe knee OA was detected among 90.9% of patients aged more than 65 years compared to 38.6% of those aged less than 40 years with detected statistical significance (p=0.017). Also, 55.7% of female patients had very/extremely severe knee OA compared to 30.4% of males (p=0.049). Out of patients, who had knee joint trauma, 65.1% complained of very/extremely severe knee OA versus 41.5% of those without (p=0.011). Additionally, 68.6% of those who took medications for osteoarthritis complained of very/extremely severe knee OA in comparison to 36% of those who did not (p=0.001). Besides, 61.9% of those who used analgesics had very/extremely severe knee OA versus 31.3% of others (p=0.001).

**Table 3:** Distribution of knee osteoarthritis severity by patients' bio-demographic data P-values are calculated utilizing Pearson's chi-squared test; ^$^ exact probability test; * p<0.05 (significant)

Factors	Osteoarthritis severity level						p-value
	Minimal/mild		Moderate/severe		Very/extremely severe		
	No	%	No	%	No	%	
Age in years							0.017*^$^
35-40	9	15.80%	26	45.60%	22	38.60%	
41-45	9	16.70%	18	33.30%	27	50.00%	
46-55	0	0.00%	0	0.00%	7	100.00%	
56-65	1	6.30%	6	37.50%	9	56.30%	
>65	0	0.00%	1	9.10%	10	90.90%	
Gender							0.049*
Male	5	21.70%	11	47.80%	7	30.40%	
Female	14	11.50%	40	32.80%	68	55.70%	
Job							0.722
No job	9	14.50%	22	35.50%	31	50.00%	
Active	8	11.80%	26	38.20%	34	50.00%	
Retired	2	13.30%	3	20.00%	10	66.70%	
Since when were you diagnosed with knee osteoarthritis?							0.711
<2 years	10	15.40%	23	35.40%	32	49.20%	
2-5 years	6	13.00%	18	39.10%	22	47.80%	
>5 years	3	8.80%	10	29.40%	21	61.80%	
Have you ever got knee joint trauma?							0.011*
Yes	4	6.30%	18	28.60%	41	65.10%	
No	15	18.30%	33	40.20%	34	41.50%	
Do you use any medications for osteoarthritis?							0.001*
Yes	3	4.30%	19	27.10%	48	68.60%	
No	16	21.30%	32	42.70%	27	36.00%	
Do you use analgesics for pain?							0.001*
Yes	6	6.20%	31	32.00%	60	61.90%	
No	13	27.10%	20	41.70%	15	31.30%	
Have you ever undergone knee replacement surgery?							0.468^$^
Yes	0	0.00%	4	44.40%	5	55.60%	
No	19	14.00%	47	34.60%	70	51.50%	
Are you visiting the physiotherapist frequently?							0.204
Yes	1	3.70%	9	33.30%	17	63.00%	
No	18	15.40%	42	35.90%	57	48.70%	

Table 4 shows the association between physical activity level and severity of knee osteoarthritis. Among males, 53.8% of patients with low physical activity had very/extremely severe knee OA compared to none of those with moderate to high physical activity levels (p=0.023). As for females, 69.9% with low physical activity levels had very/extremely severe knee OA compared to 26.7% with high physical activity levels. Totally, 67.7% of patients with low physical activity complained of very/extremely severe knee OA compared to 23.5% of those with high levels.

**Table 4:** The association between physical activity level and severity of knee osteoarthritis P-values are calculated utilizing Pearson's chi-squared test; ^$^ exact probability test; * p<0.05 (significant)

Gender	Physical activity	Osteoarthritis severity level						p-value
		Minimal/mild		Moderate/severe		Very/extremely severe		
		No	%	No	%	No	%	
Male	Low	1	7.7%	5	38.5%	7	53.8%	0.023*^$^
	Moderate	4	50.0%	4	50.0%	0	0.0%	
	High	0	0.0%	2	100.0%	0	0.0%	
Female	Low	5	6.0%	20	24.1%	58	69.9%	0.001*^$^
	Moderate	5	20.8%	13	54.2%	6	25.0%	
	High	4	26.7%	7	46.7%	4	26.7%	
Total	Low	6	6.3%	25	26.0%	65	67.7%	0.001*
	Moderate	9	28.1%	17	53.1%	6	18.8%	
	High	4	23.5%	9	52.9%	4	23.5%	

## Discussion

Knee osteoarthritis (OA) is one of the highest five reasons of disability among adults in the United States and is commonly reported in the clinical setting. Nearly 12% of Americans over the age of 60 years had symptomatic knee OA, with multifactorial effects of this illness, which are varied and significant. Physicians should address patients' worries about the proper pharmacologic and nonpharmacologic management of knee OA. One of the most announced and controversial nonpharmacologic management approaches for OA is exercise [1]. People with knee OA usually ask questions like: "Should I exercise?" and "Will exercise make my arthritis worse?". Traditional responses promote the theory that knee OA is a "wear and tear" phenomenon. According to this assumption, increased physical activity, by definition, will accelerate the degenerative process. A modern understanding of the pathophysiology of knee OA and the related pattern proposes that this understanding is incomplete, if not mistaken [13].

Knee osteoarthritis is a disabling joint disease that results in degeneration of the joint cartilage. Many studies demonstrate the risk factors and complications of knee osteoarthritis, but how physical activity impacts the osteoarthritis severity needs to be studied [6]. The current study aimed to assess the level of physical activity in patients with knee osteoarthritis. Also, it aimed to assess the impact of physical activity level on the severity of knee osteoarthritis. The study showed that less than half of the patients had knee OA for less than two years, and they used medications on a daily basis among half of them. Also, more than two-thirds used analgesics to relieve associated pain, while less than one-fifth frequently visited physiotherapists.

Regarding the level of physical activity, about two-thirds of the study cases with knee OA performed a low level of physical activity, while about 10% performed high (vigorous) physical activity. Considering the severity of knee OA, less than three-quarters (71%) of the study cases complained of severe to extremely severe OA, and 13% had minimal to mild severity of knee OA. Higher severity was reported among old-aged patients, females, patients with a history of knee trauma, and those who used medications probably due to associated pain with high severity.

The study also showed that very to extremely severe knee OA was significantly associated with a low level of physical activity among male and female patients; however, the low level of physical activity can be seen more among females because of higher body weight and less outdoor activity. Literature showed that exercise therapy for OA benefit is still doubtful which is designed and prescribed for specific therapeutic goals [13]. Persuasive evidence from more than 50 randomized controlled trials (RCTs) for patients with knee OA and 10 RCTs in hip OA included the effectiveness of land-based exercise therapy in minimizing symptoms and disabilities. In comparison to pharmacological pain killers, exercise therapy showed less efficacy, especially compared to NSAIDs, but it was more effective than acetaminophen (paracetamol) for pain control in knee OA [3,14]. Though, exercise therapy had no adverse events such as NSAIDs and acetaminophen. Fransen ​​​​​​** **et al.conducted a systematic review of hip OA and concluded that exercise therapy with better obedience to currently recognized recommendations regarding frequency, duration, and intensity showed higher efficacy in decreasing pain versus exercise therapy with poor compliance [15]. On the other hand, other studies reported that the intensity and period of the individual exercise sessions are less significant for the treatment effects [16,17]. Nevertheless, the conclusions in trials regarding the effect of intensity and duration of each session are not sufficient to precisely assess their benefit [18]. Regarding the type of exercises, many studies assessed subcategories of cases with knee OA, who showed greater improvement with one type of exercise therapy than with another [19-21]. A rising issue of the literature recommends the use of proprioceptive or balance-focused activities in the management of knee OA. Consistent sharing can positively reduce pain, balance, self-efficacy, and physical functioning [22,23]. 

On the other hand, a one-year study of thousand OA patients showed a decrease in physical function and wellness with moderate to high physical activity levels [24]. A longitudinal study was conducted in 2018 involving 121 patients with chronic knee osteoarthritis, showing the intensity of knee pain level in osteoarthritis patients linked to increased physical activity level [25]. Other studies demonstrate that pain catastrophizing is associated with a decreased level of physical activity [26,27]. A longitudinal prospective study involved 239 patients diagnosed with knee osteoarthritis ranging from the degree I to IV to assess the impact of an educational program on physical activity. This study shows that a decrease in body mass index of participants after six months and after twelve months remained; thus, routine physical exercise practice can be an important instrument for enhancing functional ability and everyday physical activity in people with knee osteoarthritis [28].

Study limitation

Even though we ensure the clarity of the questionnaire items, reporting bias and misinterpretation of some questions is a primary limitation of our study. Further, we included knee osteoarthritis patients who attended rheumatology clinics besides orthopedic clinics, which might affect the significance of the association addressed, as some patients who were diagnosed with rheumatoid arthritis were included. Besides, the issue of concern is that we conducted an analytical cross-sectional study; hence we couldn't assess the temporal link between the variables. Due to the stringent inclusion criteria we had chosen, we excluded many patients, which might affect the quantities correlation.

## Conclusions

This study revealed that physical activity, especially at high levels, was associated with lower knee OA severity, which means a beneficial effect. Most of the study patients had severe to extremely severe knee OA with low physical activity levels. It is obvious that light- to moderate-intensity physical activity had beneficial impacts on knee OA. There is significant evidence in the literature that many forms of exercise, as well as less conservative approaches, showed a positive influence on OA severity and associated impairments. Further studies with a larger sample size should be carried out.

## References

[1] Hunter DJ, Schofield D, Callander E (2014). The individual and socioeconomic impact of osteoarthritis. Nat Rev Rheumatol.

[2] GBD 2015 Disease and Injury Incidence and Prevalence Collaborators (2016). Global, regional, and national incidence, prevalence, and years lived with disability for 310 diseases and injuries, 1990-2015: a systematic analysis for the Global Burden of Disease Study 2015. Lancet.

[3] Bannuru RR, Osani MC, Vaysbrot EE (2019). OARSI guidelines for the non-surgical management of knee, hip, and polyarticular osteoarthritis. Osteoarthritis Cartilage.

[4] Cui A, Li H, Wang D, Zhong J, Chen Y, Lu H (2020). Global, regional prevalence, incidence and risk factors of knee osteoarthritis in population-based studies. EClinicalMedicine.

[5] Racunica TL, Teichtahl AJ, Wang Y (2007). Effect of physical activity on articular knee joint structures in community-based adults. Arthritis Rheum.

[6] Urquhart DM, Tobing JF, Hanna FS, Berry P, Wluka AE, Ding C, Cicuttini FM (2011). What is the effect of physical activity on the knee joint? A systematic review. Med Sci Sports Exerc.

[7] Garber CE, Blissmer B, Deschenes MR (2011). Quantity and quality of exercise for developing and maintaining cardiorespiratory, musculoskeletal, and neuromotor fitness in apparently healthy adults: guidance for prescribing exercise. Med Sci Sports Exerc.

[8] de Groot IB, Bussmann JB, Stam HJ, Verhaar JA (2008). Actual everyday physical activity in patients with end-stage hip or knee osteoarthritis compared with healthy controls. Osteoarthritis Cartilage.

[9] Manal K, Gardinier E, Buchanan TS, Snyder-Mackler L (2015). A more informed evaluation of medial compartment loading: the combined use of the knee adduction and flexor moments. Osteoarthritis Cartilage.

[10] Craig CL, Marshall AL, Sjöström M (2003). International physical activity questionnaire: 12-country reliability and validity. Med Sci Sports Exerc.

[11] Ainsworth BE, Haskell WL, Whitt MC (2000). Compendium of physical activities: an update of activity codes and MET intensities. Med Sci Sports Exerc.

[12] Lequesne MG, Mery C, Samson M, Gerard P (1987). Indexes of severity for osteoarthritis of the hip and knee: validation--value in comparison with other assessment tests. Scand J Rheumatol Suppl.

[13] Esser S, Bailey A (2011). Effects of exercise and physical activity on knee osteoarthritis. Curr Pain Headache Rep.

[14] Fransen M, McConnell S, Harmer AR, Van der Esch M, Simic M, Bennell KL (2015). Exercise for osteoarthritis of the knee: a Cochrane systematic review. Br J Sports Med.

[15] Moseng T, Dagfinrud H, Smedslund G, Østerås N (2017). The importance of dose in land-based supervised exercise for people with hip osteoarthritis. A systematic review and meta-analysis. Osteoarthritis Cartilage.

[16] Juhl C, Christensen R, Roos EM, Zhang W, Lund H (2014). Impact of exercise type and dose on pain and disability in knee osteoarthritis: a systematic review and meta-regression analysis of randomized controlled trials. Arthritis Rheumatol.

[17] Regnaux JP, Lefevre-Colau MM, Trinquart L, Nguyen C, Boutron I, Brosseau L, Ravaud P (2015). High-intensity versus low-intensity physical activity or exercise in people with hip or knee osteoarthritis. Cochrane Database Syst Rev.

[18] Bartholdy C, Warming S, Nielsen SM, Christensen R, Henriksen M (2017). Replicability of recommended exercise interventions for knee osteoarthritis: a descriptive systematic review of current clinical guidelines and recommendations. Osteoarthritis Cartilage.

[19] Bennell KL, Dobson F, Roos EM (2015). Influence of biomechanical characteristics on pain and function outcomes from exercise in medial knee osteoarthritis and varus malalignment: exploratory analyses from a randomized controlled trial. Arthritis Care Res.

[20] Knoop J, Dekker J, van der Leeden M (2013). Knee joint stabilization therapy in patients with osteoarthritis of the knee: a randomized, controlled trial. Osteoarthritis Cartilage.

[21] Lim BW, Hinman RS, Wrigley TV, Sharma L, Bennell KL (2008). Does knee malalignment mediate the effects of quadriceps strengthening on knee adduction moment, pain, and function in medial knee osteoarthritis? A randomized controlled trial. Arthritis Rheum.

[22] Lee MS, Pittler MH, Ernst E (2008). Tai chi for osteoarthritis: a systematic review. Clin Rheumatol.

[23] Wang C, Schmid CH, Hibberd PL, Kalish R, Roubenoff R, Rones R, McAlindon T (2009). Tai Chi is effective in treating knee osteoarthritis: a randomized controlled trial. Arthritis Rheum.

[24] Rodrigues da Silva JM, de Rezende MU, Spada TC (2017). Educational program promoting regular physical exercise improves functional capacity and daily living physical activity in subjects with knee osteoarthritis. BMC Musculoskelet Disord.

[25] Lazaridou A, Martel MO, Cornelius M (2019). The association between daily physical activity and pain among patients with knee osteoarthritis: the moderating role of pain catastrophizing. Pain Med.

[26] Bousema EJ, Verbunt JA, Seelen HA, Vlaeyen JW, Knottnerus AJ (2007). Disuse and physical deconditioning in the first year after the onset of back pain. Pain.

[27] Estévez-López F, Álvarez-Gallardo IC, Segura-Jiménez V (2018). The discordance between subjectively and objectively measured physical function in women with fibromyalgia: association with catastrophizing and self-efficacy cognitions. The al-Ándalus project. Disabil Rehabil.

[28] Rogers LQ, Macera CA, Hootman JM, Ainsworth BE, Blair SN (2002). The association between joint stress from physical activity and self-reported osteoarthritis: an analysis of the Cooper Clinic data. Osteoarthritis Cartilage.

